# Enhanced oral nanomedicine utilizing biomineralized oncolytic virus for synergistic gastrointestinal cancer therapy

**DOI:** 10.1016/j.mtbio.2025.102583

**Published:** 2025-11-21

**Authors:** Zujian Hu, Yining Sun, Shenlei Yu, Fan Zheng, Zhuo Yan, Ning Lu, Luyi Ye, Shanshan Yuan, Yuting Zhu, Junjie Deng, Jilong Wang, Yongheng Bai

**Affiliations:** a Zhejiang Key Laboratory of Intelligent Cancer Biomarker Discovery and Translation, The First Affiliated Hospital, Wenzhou Medical University, Wenzhou, 325035, China; b National Clinical Research Center for Ocular Diseases, Eye Hospital, Wenzhou Medical University, Wenzhou, 325027, China; c Joint Centre of Translational Medicine, The First Affiliated Hospital of Wenzhou Medical University, Wenzhou Medical University, Wenzhou, Zhejiang, 325000, China; d Joint Centre of Translational Medicine, Wenzhou Institute, University of Chinese Academy of Sciences, Wenzhou, Zhejiang, 325000, China

**Keywords:** Oncolytic adenoviruses, Gastrointestinal tumor, Biomineralized, Cancer therapy

## Abstract

Oncolytic viruses (OVs) represent a promising nanomedicine strategy for cancer therapy, yet their clinical application-particularly *via* oral administration-remains challenging due to degradation by digestive enzymes and neutralization by antibodies in the gastrointestinal tract. To address this, we developed a biomineralized cancer membrane-coated oncolytic adenovirus (CaCO_3_@CM-OA) with enhanced resistance to enzymatic and immune clearance, significantly improving tumor-targeting efficiency. In colorectal and pancreatic cancer models, this engineered virus induced potent anti-tumor effects *via* G2/M phase arrest, mediated by p53 phosphorylation and p21 upregulation, while suppressing epithelial-mesenchymal transition (EMT) through downregulation of N-cadherin, vimentin, and α-SMA. Furthermore, the virus triggered multimodal regulated cell death, including mitochondrial apoptosis, autophagy, and necroptosis, accompanied by immunogenic cell death (ICD) markers such as ATP release, calreticulin exposure, and HMGB1 translocation, indicating robust immune activation. Transcriptomic analysis further revealed downregulation of pro-survival genes (e.g., RHBDD2) and modulation of proliferation-related (e.g., ZMYND10, CDC27, ST7) and endocytosis-related (SNX11) genes, elucidating its multifaceted mechanism. This study highlights the potential of biomineralized OVs to overcome oral delivery barriers and enhance therapeutic efficacy in gastrointestinal cancers. By inducing synergistic cell death and immune activation, this strategy provides a foundation for clinical translation and identifies novel molecular targets for future investigation. Our findings underscore the feasibility of engineered oral OV formulations to improve treatment outcomes in intestinal malignancies.

## Graphical abstract

**Figure:**
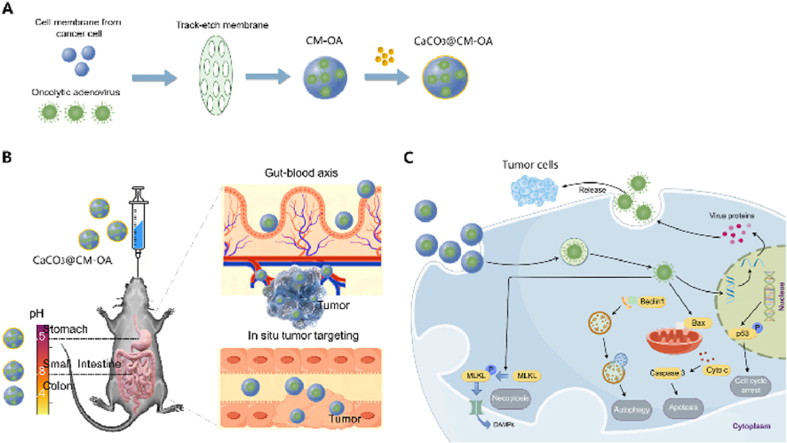

## 1 Introduction

Oncolytic viruses (OVs) have emerged as a groundbreaking class of cancer therapeutics, with numerous candidates demonstrating promising efficacy in preclinical and clinical trials [1,2]. These genetically engineered or naturally occurring viruses selectively infect and lyse tumor cells while stimulating systemic antitumor immunity, offering a dual mechanism of action that distinguishes them from conventional therapies [[3], [4], [5], [6], [7]]. To date, multiple OV platforms-including adenoviruses (e.g., ONYX-015), herpes simplex viruses (e.g., T-VEC), and vaccinia viruses (e.g., Pexa-Vec)-have advanced to clinical testing, with T-VEC achieving FDA approval for melanoma treatment [[8], [9], [10]]. Beyond direct oncolysis, OVs remodel the tumor microenvironment by releasing tumor-associated antigens and danger signals [11], thereby enhancing immune checkpoint blockade and adoptive cell therapies. Despite these advances, the clinical application of OVs remains constrained by significant delivery challenges. Systemic administration, while feasible for some malignancies, is often hindered by rapid viral clearance, neutralizing antibodies, and off-target toxicity, particularly hepatotoxicity [12,13]. Intratumoral injection, though effective for accessible lesions, is impractical for disseminated or deep-seated tumors, such as pancreatic or metastatic colorectal cancers [14]. These limitations are exacerbated in gastrointestinal (GI) malignancies, where anatomical barriers further restrict therapeutic access.

Oral delivery of OVs presents a transformative strategy to overcome these hurdles, particularly for GI cancers. The gastrointestinal tract offers direct anatomical access to primary and metastatic lesions, enabling localized viral replication while minimizing systemic exposure. In addition, the sustained-release properties of oral administration can provide prolonged viral exposure within the tumor microenvironment [15]. However, the harsh GI environment-characterized by acidic pH, digestive enzymes, and mucosal barriers-has traditionally precluded the use of unprotected OVs [16].

Recent advances in nanotechnology now offer innovative solutions to these challenges, with biomineralization and cell membrane coating emerging as particularly promising strategies to protect and target OVs for oral delivery. Biomineralization-particularly with calcium carbonate (CaCO_3_)-provides a robust platform for oral formulations. CaCO_3_ nanoparticles exhibit pH-responsive dissolution, remaining stable in the acidic stomach environment while releasing their payload in the neutral-to-alkaline intestinal tract, where GI tumors are often localized. This pH-dependent release profile minimizes premature payload degradation. Additionally, CaCO_3_ nanoparticles can transiently modulate intestinal tight junctions, enhancing payload penetration across the epithelial barrier. Their porous structure allows high payload loading, while the mineral shell protects payload from digestive enzymes and bile salts [[17], [18], [19]]. Cell membrane coating technology further enhances oral payload delivery by addressing immune evasion and targeted homing [20,21]. Coating payload with cancer cell membranes (CCMs) confers "self-recognition" properties, enabling homologous targeting to residual or metastatic tumor cells through preserved adhesion molecules [22]. CCMs also shield payload of OV from neutralization by preexisting antibodies and complement activation, extending circulation time [23]. The combination of biomineralization and membrane coating creates a synergistic delivery system: the CaCO_3_ core protects against biochemical degradation, while the membrane cloak facilitates immune evasion and tumor targeting.

Herein, to address the critical challenges of oral oncolytic virotherapy, we engineered a biomineralized, cancer cell membrane-coated oncolytic adenovirus (CaCO_3_@CM-OA) through a multi-step bioinspired strategy. First, the oncolytic adenovirus was cloaked with homologous cancer cell membranes (CM-OA) derived from target tumors, endowing the construct with immune-evading properties and tumor-specific tropism. Subsequently, the CM-OA was decorated with the CaCO_3_ via the co-precipitation, which could provide pH-responsive protection against gastric degradation while enabling intestinal release. In vitro and in vivo characterization revealed that CaCO_3_@CM-OA exhibited remarkable resistance to enzymatic and immune clearance with higher tumor accumulation than unmodified OA after oral administration. Mechanistically, the engineered virus induced G2/M cell cycle arrest via p53/p21 activation and suppressed epithelial-mesenchymal transition (EMT) by downregulating N-cadherin, vimentin, and α-SMA. Transcriptomic profiling identified the concurrent modulation of proliferative (e.g., ZMYND10, CDC27) and endocytic (SNX11) pathways, alongside induction of multimodal cell death (apoptosis, necroptosis, and autophagy) and immunogenic cell death (ICD). These findings establish CaCO_3_@CM-OA as a versatile platform for overcoming oral delivery barriers while amplifying therapeutic efficacy through synergistic oncolysis and immune activation in gastrointestinal cancers.

## 2 Methods

### 2.1 Drugs and antibodies

The antibodies used in western blot, immunofluorescence and immunohistochemistry experiments were as follows: CRT (Bioss, 5913R), HMGB1 (Beytime, AF1174), Anti-Adenovirus Type 5 Hexon (abcam, ab816352), Ki67 (Proteintech, 27309-1-AP), p53 (MCE, HY-P80938), p-p53 (Affinity, AF3075), p21 (Affinity, AF6290), CyclinB1 (Proteintech, 28603-1-AP), Bcl2 (Proteintech, 26593-1-AP), Bax (Proteintech, 50599-2-Ig), Caspase3 (Proteintech, 19677-1-AP), cleaved-Caspase3 (CST, 9661S), Cytochrome *c* (Proteintech, 10993-1-AP), p62 (HuaBio, HA721171), Beclin1 (CST, 3495S), LC3A/B (CST, 12741T), p-MLKL (MCE, HY-P81878), MLKL (HuaBio, ET1601-25), N-cadherin (Affinity, AF5239), E-cadherin (Affinity, AF0131), α-SMA (HuaBio, ET1607-53), Vimentin (Affinity, AF7013), Collegen-I (Proteintech, 66761-1-Ig), Collegen-III (Abcam, ab7778).

### 2.2 Cell culture and treatments

Pancreatic cancer cell line PaTu-8988t, colorectal cancer cell line HT29, embryonic kidney cell line HEK293 and fibroblast cell line L929 were obtained from the Chinese Academy of Sciences Cell Bank (Shanghai, China). Cells were cultured in Dulbecco's modified Eagle medium (DMEM) supplemented with 10 % fetal bovine serum (Gibco, USA) and 1 % antibiotic under conditions of 5 % CO_2_ and 37 °C in an incubator. HT29 and PaTu-8988t cells were incubated with OA (MOI = 1 × 10^2^), CM-OA (MOI = 1 × 10^2^), or CaCO_3_@CM-OA (MOI = 1 × 10^2^) for 48 h.

### 2.3 Animals and treatments

Male BALB/c nude mice (5-weeks) were acquired from Zhejiang Province Experimental Animal Center. All mice were bred and housed in SPF environment. All animal experiments were approved by the Animal Care and Use Committee of Wenzhou Institute, University of Chinese Academy of Sciences (WIUCAS24053102).

As previous describe [24], to induce orthotopic colorectal tumors, 50 μL of HT29 cells (1 × 10^6^) in PBS were injected into colorectal wall of BALB/c nude mice. After giving a light pressure to injection site to avoid leakage, the cecum was returned to the peritoneal cavity and the incision was sutured. After 10 days, 24 mice were randomly divided into four groups (n = 6 in each group).

For the pancreatic cancer model [25], PaTu-8988t (1 × 10^6^) in 0.2 ml PBS was subcutaneously injected into the right armpit of nude mice to establish nude mouse subcutaneous model. 3 weeks after inoculation, when the size of the tumor reached 100 mm^3^, 24 mice were randomly divided into four groups (n = 6 in each group).

### 2.4 Preparation of cell membrane

When HT29 cells and Patu-8988t cells were cultured to 70–80 % confluence, cells were digested with trypsin and collected for subsequent lysis operations. In order to preserve the structural and functional integrity of the cell membrane, Tris, mannitol, EDTA-Na2 and sucrose dissolved in PBS buffer was act as mild lysis buffer. Cell membranes were ultimately obtained after three centrifugations at 2000 g, 4 °C for 10 min each, followed by one centrifugation at 21,000 g, 4 °C for 30 min.

### 2.5 Preparation and characterization of CaCO_3_@CM-OA

Firstly, in order to obtain membrane vesicles, cell membrane suspension was extruded through a 5000 nm and 1000 nm polycarbonate track-etch membrane (Merck, Darmstadt, Germany) for at least 11 cycles. Subsequently, to prepare oncolytic adenovirus (Fubio, OVAD199-2282) encapsulated with membrane vesicles (CM-OA), we extruded a mixture of membrane vesicles and oncolytic adenovirus through a 200 nm polycarbonate track-etched membrane for at least 11 cycles. Following this, a calcium carbonate shell was formed on the surface of CM-OA via biomimetic mineralization. Specifically, the CM-OA suspension was mixed with cyclohexane, 1-hexanol, and Triton X-100 in a volume ratio of 3:7.5:1.8:1.77. Then, 800 μL of calcium chloride (30 mM) was added, and the mixture was stirred at 700 rpm for 30 min using a magnetic stirrer. Afterward, 180 μL of sodium carbonate (2.92 mM) was introduced, and the stirring continued for 6–10 h to obtain biomimetic mineralized oncolytic adenovirus (CaCO_3_@CM-OA).

The particle size and zeta potential of OA, CM-OA and CaCO_3_@CM-OA was determined by a ZEN3600 particle size analyzer (Malvern, UK). The morphology of CaCO_3_@CM-OA was observed using a scanning electron microscopy (SU8010, Hitachi, Japan) and a transmission electron microscope (Talos-F200S, Thermo Fisher, USA), and the elemental analysis was performed by X-ray photoelectron spectroscopy (XPS, Thermo Fisher, USA).

### 2.6 Silver staining

According to manufactory instruction, membrane vesicles proteins were resolved on 10 % SDS-PAGE gels and then visualized by Fast Silver Stain Kit (P0017S; Beyotime, China). In briefly, after electrophoresis and fixation, gels were successively placed into sensitization solution, sliver solution, silver dye color developing solution and finally the reaction was terminated by stop solution. Images was taken and applied to protein identification.

### 2.7 Dot blot analysis

Dot blot analysis was performed to determinate coat protein of naked OA, CM-OA and CaCO_3_@CM-OA. As previously described, 10ul sample of OA, CM-OA or CaCO_3_@CM-OA (10^4^ GFU/ml) was blotted onto PVDF membrane and fixed by drying 20min at room temperature. Then membrane was blocked with 5 % skim milk for 2 h followed by incubation with the antibody of Anti-Adenovirus Type 5 Hexon at 4 °C overnight. The membrane was washed three times in Tris-buffered saline with 0.1 % Tween 20 (TBST) the following day and incubated with HRP-conjugated anti-rabbit antibody. Finally, the blot was detected by enhanced chemiluminescence (ECL) and visualized by autoradiography.

### 2.8 Cell viability assay

To evaluate cytotoxicity of CaCO_3_@CM-OA, a cell counting kit-8 (CCK-8) assay was performed. Firstly, HT29, Patu-8988t and NCM460 cells were cultured in 96-well plates at a density of 5 × 10^3^ cells per well. Next, the same virus load of OA, CM-OA and CaCO_3_@CM-OA (MOI = 10) were suspended and incubated in simulated gastric juice at 37 °C for 2h before co-culture with cells [26]. After co-culture for 48 h, 10 μL of CCK-8 solution for 2 h to detect the absorption value at 450 nm with a microplate reader and calculate the relative cell viability of each well.

### 2.9 Colony formation assay

In order to determine the proliferation ability of cells, colony formation assay was performed as previous described. In briefly, HT29 and Patu-8988t cells were seeded in six-well plates with the density of 800 cells per well. After 48 h treatment of PBS (MOI = 0), OA (MOI = 10), CM-OA (MOI = 10), or CaCO_3_@CM-OA (MOI = 10), change to normal medium and keep culturing for 7day. Then, colonies were fixed with 4 % paraformaldehyde for 30min. Finally, cell colonies were stained with crystal violet and counted. Experiments were independently performed at least three times.

### 2.10 Wound healing assay

Wound healing assay was carried out to evaluate cell migration. Briefly, HT29 and Patu-8988t cells were seeded in six-well plates at a concentration of 2 × 10^5^ cell per well and, after confluence reaching 100 %. A scratch was introduced on the cell monolayer with a 200 μl sterile pipet tip. The detached cells were washed with PBS. Then the medium containing PBS (MOI = 0), OA (MOI = 10), CM-OA (MOI = 10), or CaCO_3_@CM-OA (MOI = 10) was added. Images of scratch were taken with an invert microscope ever 24 h. Image J software (Version 1.48, NIH, USA) and GraphPad Prism software (v8.3.1, USA) were used to calculate the scratch healing rate. The experiments were repeated more than three times.

### 2.11 Transwell invasion assay

The transwell invasion assay was performed to measure the invasion ability of HT29 and Patu-8988t cells. The gel-coated 24-well plates in the chamber were purchased from Costar (8 μm, Costar, Corning, USA). HT29 and Patu-8988t cells were planted into the upper chamber in the 200 μL serum-free culture medium which respectively containing PBS (MOI = 0), OA (MOI = 10), CM-OA (MOI = 10), or CaCO_3_@CM-OA (MOI = 10) at the density of 5 × 10^4^ cells/well. Meanwhile, 600 μL culture medium with 20 % FBS was added to the lower chamber of each well. After 48 h incubation, the cells were fixed with 4 % paraformaldehyde and stained with 0.5 % crystal violet. Finally, images of cells invaded to the lower side of the chamber were captured, and more than six different fields were randomly selected for counting. All the experiment were performed in triplicate.

### 2.12 Apoptosis analysis

According to manufacturer's instructions, Annexin V-APC/PI apoptosis kit (E-CK-A217, Elabscience, USA) was applied to measure apoptosis rate of cells. Firstly, HT29 and Patu-8988t cells were seeded at 5 × 10^4^ cells per well in a 24-well plate and then respectively treated with PBS (MOI = 0), OA (MOI = 10), CM-OA (MOI = 10), or CaCO_3_@CM-OA (MOI = 10) for 48 h as previous description. Afterward, cells were harvested and stained with APC-labeled Annexin V and PI for 20 min shielded from light at 4 °C. Finally, the apoptosis rate of cells was analyzed by CytoFLEX flow cytometer (Beckman Coulter, Brea, CA, USA).

### 2.13 Quantitative real-time qRT-PCR

Total RNA from cells and tissues was extract by trizol reagent (GLPBIO, Shanghai, China), following the manufacturer's instructions. Then 1 μg of total RNA was reverse transcribed to synthetize the cDNA template by HiScript III RT SuperMix for qPCR kit (Vazyme, Nanjing, China). The resulting cDNA act as template to perform quantitative PCR using the Vazyme SYBR Green Mix Kit. The qRT-PCR reaction was performed on an a QuantiStudio 5 System (Thermo Fisher, USA). The ΔΔCt method was used for calculations of the relative expression levels.

### 2.14 Western blot analysis

As previously described, total protein of cells and tissues were extracted by RIPA (Beyotime, Shanghai, China). Then, the total protein was purified by 12 % SDS-PAGE and transferred to PVDF membranes. Next, the membrane was blocked by 5 % skim milk and incubated with primary antibodies. After incubation overnight at 4 °C, the membrane was hybridized with secondary antibody for 1 h at room temperature. Finally, protein bands were visualized via chemiluminescence reagents (ECL, A38554, Thermo Fisher Scientific) on autoradiographic film.

### 2.15 Real-time cellular analysis (RTCA)

For monitoring proliferation and viability of cells, HT29 and Paru-8988t cells were seeded in a 16-well E-Plates (Agilent, USA) at 8 × 10^3^ cells/well. After reaching a stable growth state, cells were treated with PBS, OA, CM-OA, or CaCO_3_@CM-OA as described previously. xCELLigence RTCA MP System (ACEA Biosciences, San Diego, CA, USA) was applied to generate Cell index curves.

### 2.16 Immunofluorescence staining

Immunofluorescence staining was performed as previously described. In briefly, HT29 and Patu-8988t cells were inoculated in 24-well plates at 5 × 10^4^ cells/well for incubation with PBS, OA, CM-OA, or CaCO_3_@CM-OA. After 48 h, cells were fixed with 4 % paraformaldehyde (Solarbio) for 30 min and permeabilized using 0.1 % Triton X-100 (Solarbio) for 10 min. Then, slides of cells were blocked by normal goat serum and incubate with primary antibody overnight. The next day, the slides were incubated with a fluorescent secondary antibody for 1 h at 37 °C (in the dark), and the nuclei were stained with DAPI.

### 2.17 Histological examination and immunohistochemical staining

After fixed in 4 % paraformaldehyde, tumor tissues from animals were paraffinized and sliced into sections with 4 μm thickness. Next, procedures of deparaffinization and hydration were performed. For HE staining, sections were stained with hematoxylin and eosin (Solarbio). For immunohistochemical analysis, sections were treated with 0.1 % sodium citrate to repair antigen and incubate with 3 % H_2_O_2_ to block endogenous peroxidase. Then, non-specific binding sites of sections were blocked by 5 % normal goat serum, and primary antibody were added for incubation overnight. The next day, sections were incubated with corresponding secondary antibodies, and stained by DAB Horseradish Peroxidase Color Kit.

### 2.18 Statistical analysis

The data are represented as the mean ± SD. GraphPad Prism 9.0 software was utilized for statistical analyses, with one-way ANOVA followed by the Tukey's post hoc test to evaluate intergroup differences. A p-value that is less than 0.05 was deemed to be statistically significant.

## 3 Results

### 3.1 Physicochemical analysis of CaCO_3_@CM-OA

**Fig. 1:**
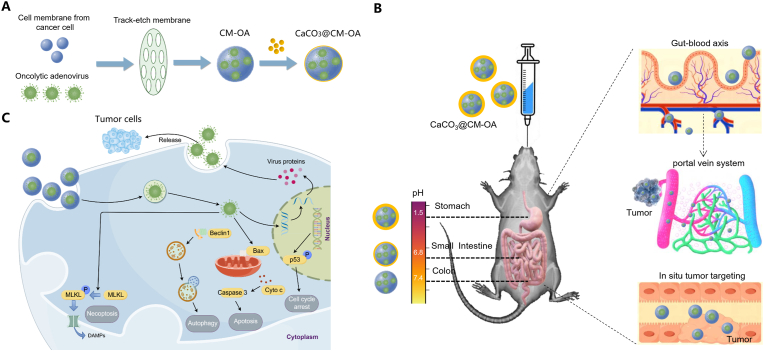
Schematic illustration of CaCO_3_@CM-OA. (A) Schematic representation of CaCO_3_@CM-OA preparation. (B) CaCO_3_@CM-OA delivers OA to tumor sites through the gastrointestinal tract and systemic circulation. (C) Upon infection of tumor cells, OA induces the activation of multiple cell death signaling pathways.

**Fig. 2:**
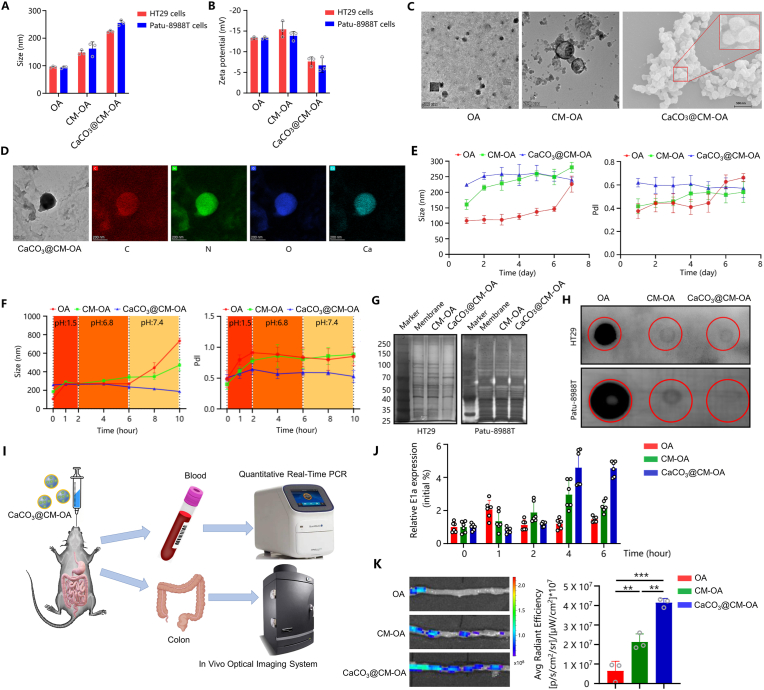
Construction and characterization of CaCO_3_@CM-OA. (A) Particle size analysis of OA, CM-OA, and CaCO_3_@CM-OA. (B) Zeta potential measurements of OA, CM-OA, and CaCO_3_@CM-OA. (C) TEM image of OA and CM-OA, together with SEM image of CaCO_3_@CM-OA. (D) TEM image and corresponding elemental mapping of CaCO_3_@CM-OA. (E, F) Size and PDI of OA, CM-OA, and CaCO3@CM-OA under different pH conditions and time points. (G) Silver-stained SDS-PAGE gel comparing cell membrane, CM-OA, and CaCO_3_@CM-OA. (H) Dot blot assay detecting adenovirus type 5 hexon in OA, CM-OA, and CaCO_3_@CM-OA. (I) Blood and intestinal tissues were collected from mice after oral administration. (J) Relative viral levels in blood at various time points and (K) relative fluorescence intensity in intestinal tissues were quantified. Data are presented as mean ± SD. ∗P < 0.05, ∗∗P < 0.01, ∗∗∗P < 0.001.

CaCO_3_@CM-OA was constructed through a two-step process: Firstly, the oncolytic adenovirus was encapsulated using tumor cell membranes; Secondly, CaCO_3_ mineralization was applied on the surface of cell membranes (Fig. 1A). CaCO_3_@CM-OA can directly reach colorectal tumor lesions through the digestive tract or enter the bloodstream via the submucosal capillaries of the intestine, ultimately arriving at distant lesions (Fig. 1B). Upon reaching the lesion, CaCO_3_@CM-OA creates a high calcium ion concentration environment around tumor cells, enhancing their ability to uptake viral particles. The oncolytic adenovirus exerts its antitumor effects through multiple mechanisms upon infecting tumor cells (Fig. 1C). We evaluated the morphological and stability characteristics of the constructed CaCO_3_@CM-OA. Initially, we used a particle size analyzer to measure the particle size and zeta potential of CaCO_3_@CM-OA prepared with HT29 or Patu-8988T cell membranes (Fig. 2A and B). The results showed that the prepared CaCO_3_@CM-OA had a size range of 240–270 nm, and compared to OA and CM-OA, the surface zeta potential of CaCO_3_@CM-OA was reduced, indicating successful coating of the mineralized shell on the surface. Subsequently, scanning electron microscopy (SEM) was utilized to evaluate the morphological features of CaCO_3_@CM-OA (Fig. 2C). Additionally, transmission electron microscopy (TEM) was applied to performed elemental analysis. The distribution of C, N, O, and Ca elements further confirmed the successful construction of CaCO_3_@CM-OA (Fig. 2D). Additionally, we conducted assessments of the storage stability (Fig. 2E) and pH stability (Fig. 2F) of OA, CM-OA, and CaCO_3_@CM-OA under specific conditions. The results indicated that, compared to OA and CM-OA, CaCO_3_@CM-OA exhibited relatively stable particle size and PdI over a period of 7 days. Furthermore, there were no drastic changes in particle size under acidic conditions, demonstrating a good stability. To investigate whether the mineralization modification affects the protein profiles of the tumor cell membrane, we performed gel electrophoresis (SDS-PAGE) followed by rapid silver staining for visualization. The results showed that the preparation of CaCO_3_@CM-OA had no or little effect on protein profiles of the cell membrane (Fig. 2G). Furthermore, dot blot and immunofluorescence analysis suggested that the immune evasion function provided by the tumor cell membrane was preserved (Fig. 2H–S1). Meanwhile, as shown in Fig. 2I, mice were administered with CaCO_3_@CM-OA via gavage, and the relative virus content in the serum was detected at 0, 1, 2, 4, and 6 h (Fig. 2J). The results indicated that CaCO_3_@CM-OA could prolong the circulation time of the oncolytic adenovirus in the blood compared to OA and CM-OA. Ex vivo imaging of the intestine also suggested better intestinal retention of CaCO_3_@CM-OA (Fig. 2K).

### 3.2 CaCO_3_@CM-OA enhances tumor cell targeting and infectivity of oncolytic adenovirus

**Fig. 3:**
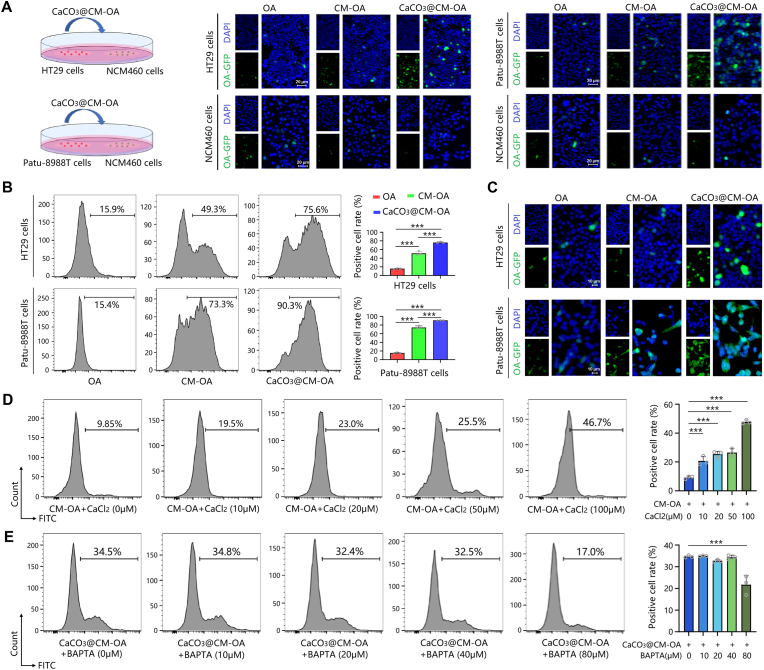
CaCO_3_@CM-OA enhances OA infectivity in tumor cells. (A) Co-cultures of HT29 or Patu-8988T with NCM460 cells were treated with OA, CM-OA, or CaCO_3_@CM-OA. Infected cells display green fluorescence. (B) Flow cytometry analysis of infection efficiency in HT29 and Patu-8988T cells. (C) Microscopy images showing green fluorescence in infected HT29 and Patu-8988T cells. (D) Flow cytometry of HT29 and Patu-8988T cells treated with different concentrations of CaCl_2_, evaluating uptake of CM-OA. (E) Flow cytometry of cells treated with various concentrations of BAPTA, assessing CaCO_3_@CM-OA uptake. Data are presented as mean ± SD. ∗P < 0.05, ∗∗P < 0.01, ∗∗∗P < 0.001.

The key factors limiting the efficacy of oncolytic adenovirus therapy are tumor-specific targeting and infectivity. To investigate the targeting efficiency of viral particles, HT29 or Patu-8988T cells were co-cultured with normal intestinal epithelial NCM460 cells, respectively. The results indicated that the modification of the extracellular tumor cell membrane could endow the oncolytic adenovirus with certain tumor-targeting capabilities (Fig. 3A). The difference between CaCO_3_@CM-OA and CM-OA lies in the presence of a calcium carbonate shell in CaCO_3_@CM-OA. Under simulated conditions, we found that the presence of this shell not only did not reduce the proportion of infected tumor cells, but enhanced the infectivity of the virus (Fig. 3B and C). To further investigate whether calcium ions contribute to the enhanced uptake capacity of tumor cells, we altered the calcium ion content around the tumor cells by calcium chloride and the extracellular calcium ion chelator BATPA. After incubation with CM-OA or CaCO_3_@CM-OA, the tumor cell infection rate was detected by flow cytometry. As shown in Fig. 3D and E, the local calcium ions provided by the calcium carbonate shell might benefit to the elevated infection.

### 3.3 CaCO_3_@CM-OA inhibits tumor proliferation, migration, and invasion

**Fig. 4:**
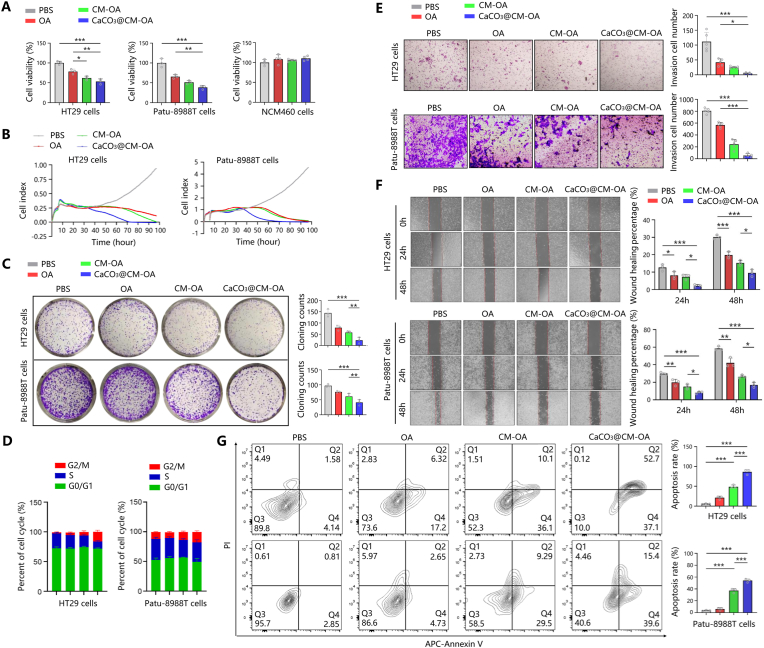
CaCO_3_@CM-OA inhibits tumor cell proliferation and metastasis while inducing apoptosis. (A) CCK-8 assay evaluating oncolytic activity. (B) Cell viability assessed via RTCA. (C) Colony formation assay demonstrating proliferation. (D) Flow cytometry showing increased G2/M arrest upon CaCO_3_@CM-OA treatment. (E) Transwell assays and quantification of HT29 and Patu-8988T migration. (F) Wound healing assays. (G) Apoptosis rates measured in HT29 and Patu-8988T cells. Data are presented as mean ± SD. ∗P < 0.05, ∗∗P < 0.01, ∗∗∗P < 0.001.

Malignant proliferation is a significant characteristic of tumor. Firstly, we investigated the effects of PBS, OA, CM-OA, and CaCO_3_@CM-OA treatments on the viability of HT29 cells and Patu-8988T cells by CCK-8 assay. The results reveal that CaCO_3_@CM-OA treatment inhibited over 50 % of both tumor cell types, while having no significant impact on the viability of normal intestinal epithelial NCM460 cells (Fig. 4A). This suggests that CaCO_3_@CM-OA can inhibit tumor cell proliferation and prevent normal cells from damage. Subsequently, an RTCA experiment was conducted to monitor the growth status of cells treated with PBS, OA, CM-OA, or CaCO_3_@CM-OA over 96 h. The growth curves also indicated that tumor cells treated with CaCO_3_@CM-OA showed earlier and more significant inhibition of proliferation (Fig. 4B). In addition, colony formation experiments demonstrated that the number of clone formation in CaCO_3_@CM-OA treated cancer cells significantly decreased, compared to control groups (Fig. 4E).

Metastasis is another crucial feature of malignant tumors. Specifically, tumor cells lose their epithelial characteristics and can break through the primary tumor site and spread to other parts of the body. Therefore, to evaluate the metastasis ability of tumor cells with different treatments, we conducted a scratch assay by co-incubating tumor cells with PBS, OA, CM-OA, or CaCO_3_@CM-OA, respectively. The results showed that CaCO_3_@CM-OA exhibited a good inhibitory effect on the migration rate of HT29 and Patu-8988T cells after 48 h of co-incubation (Fig. 4F). We then examined the impact of CaCO_3_@CM-OA on the invasive ability of HT29 and Patu-8988T cells. As indicated by the results, CaCO_3_@CM-OA effectively reduce the number of HT29 and Patu-8988T cells penetrate from the Transwell upper chamber to the lower chamber (Fig. 4C). These results suggest that CaCO_3_@CM-OA can effectively inhibit the metastasis and invasive ability of colorectal and pancreatic cancers.

### 3.4 CaCO_3_@CM-OA induces tumor cell cycle arrest at G2/M checkpoint

To further investigate the effect of CaCO_3_@CM-OA on tumor proliferation, we determined the mRNA levels of Ki67 in each group via qRT-PCR. The results revealed that the treatment of CaCO_3_@CM-OA significantly reduced the transcript level of Ki67 compared to control groups (Fig. S3A). Western blot analysis showed a similar trend in HT29 and Patu-8988T cells at the protein level (Fig. S3B). Additionally, immunofluorescence experiments indicated the intensity of Ki67 fluorescence significantly weakened in both cell types after CaCO_3_@CM-OA treatment (Fig. S3C). Subsequently, we examined the expression of cell cycle-related proteins such as P21 and cyclin B1. The results demonstrated that CaCO_3_@CM-OA led to an upregulation of p21 and p-p53, as well as the accumulation of cyclin B1, suggesting cell cycle arrest as a result of treatment (Fig. S3D). Furthermore, flow cytometry analysis (Fig. 4D and Fig. S3E) showed an increased proportion of cells in the G2/M phase after CaCO_3_@CM-OA treatment, indicating the cell cycle arrest occurred in G2/M phase.

### 3.5 CaCO_3_@CM-OA inhibits epithelial-mesenchymal transition in tumor cells

Epithelial-mesenchymal transition (EMT) is a critical mechanism in tumor metastasis. Through the EMT process, epithelial cells lose their original polarity and intercellular connections, acquiring the characteristics of mesenchymal cells. This transformation enables tumor cells to exhibit enhanced migratory and invasive capabilities, thus facilitating tumor metastasis. To evaluate the effects of different treatments on the EMT process in tumor, we treated tumor cells with PBS, OA, CM-OA, and CaCO_3_@CM-OA. Following treatment, the mRNA expression levels of EMT-related markers in HT29 cells, including N-cadherin, Vimentin, and TGF-β were assessed. The results indicated that CaCO_3_@CM-OA significantly inhibited the expression of these markers compared to control groups such as OA. Similarly, Patu-8988T cells treated with CaCO_3_@CM-OA showed comparable changes (Fig. S4A). As illustrated in Fig. S4B, at the protein expression level, CaCO_3_@CM-OA treatment resulted in a more pronounced downregulation of N-cadherin, Vimentin, and Collagen-1 compared to the PBS, OA, and CM-OA groups. Immunofluorescence staining also revealed a reduced intensity of Vimentin staining in HT29 and Patu-8988T cells after incubation with CaCO_3_@CM-OA (Fig. S4C).

### 3.6 Apoptotic and autophagic cell death induction by engineered CaCO_3_@CM-OA

Resistance to apoptosis is the characteristic of malignancy, so the induction of apoptosis was referred as an effective strategy for tumor treatment. Firstly, we evaluated the cell apoptosis rate in HT29 and Patu-8988T cells treated with PBS, OA, CM-OA, or CaCO_3_@CM-OA by flow cytometry analysis (Fig. 4G). The results confirmed that CaCO_3_@CM-OA exhibits stronger apoptosis-inducing capability compare to OA and CM-OA. Examination of Bcl2 and Bax in mRNA expression level reveals that CaCO_3_@CM-OA significantly upregulated Bax levels while decreasing Bcl2 expression (Fig. S5A). Additionally, the expression of cytochrome *c* and cleaved-Caspase 3 proteins in HT29 and Patu-8988T cells was significantly elevated after CaCO_3_@CM-OA treatment (Fig. S5B and C). Immunofluorescence analysis of Bcl2 showed a reduction of fluorescence intensity (Fig. S5D), indicating enhanced apoptotic signaling in colorectal cancer and pancreatic cancer cells upon CaCO_3_@CM-OA exposure.

Autophagy is a normal physiological process that degrades and recycles proteins or organelles when cells encounter abnormal stimuli. Although autophagy serves as a cellular self-protection mechanism, autophagy induced by viral infection is not always beneficial to cells. Studies have demonstrated that oncolytic viruses exert antitumor effects through autophagy in two ways: Firstly, viruses can hinder autophagy-inhibiting signals such as AKT/mTOR, leading to increased autophagosome formation, subsequent membrane damage, and promotion of tumor cell death. Secondly, viruses can utilize autophagosomes as replication sites, thereby enhancing oncolytic virus replication. These two aspects exhibit a positive feedback effect, amplifying the oncolytic activity of the virus. By assessing the expression of autophagy pathway markers, we found that OA, CM-OA, and CaCO_3_@CM-OA can induce autophagy in tumor cells to varying degrees. Specifically, this was manifested by a decrease in p62 protein levels and an increase in Beclin 1 and LC3B/LC3A (Fig. S6A).

### 3.7 Engineered CaCO_3_@CM-OA induces necrosis and immunogenic cell death in tumors

Previous studies have demonstrated that necroptosis, distinct from other programmed cell death like apoptosis, can occur concurrently with viral infection. Necroptosis is characterized by its independence from caspase activity and primarily relies on the phosphorylation of MLKL. Once phosphorylated, MLKL oligomerizes and assembles into pore complexes on the cell membrane, increasing membrane permeability and releasing damage-associated molecular patterns (DAMPs). This process renders necroptosis a highly immunogenic form of cell death.

In our investigation, we initially measured the levels of lactate dehydrogenase (LDH) in the cell culture supernatants of various groups (Fig. S6D), revealing a significant release of LDH following CaCO_3_@CM-OA treatment. Subsequently, as shown in Fig. S6B, CaCO_3_@CM-OA treatment effectively induced the expression of p-MLKL in both HT29 and Patu-8988T cells. And immunofluorescence results further supported this finding, showing the aggregation of p-MLKL at the membrane (Fig. S6C).

To further investigate the induction of multiple cell death modalities by CaCO_3_@CM-OA as previous described, we co-cultured tumor cells with CaCO_3_@CM-OA in the presence of necroptosis inhibitor Nec-1, apoptosis inhibitor Z-VAD, or autophagy inhibitor CQ. CCK-8 assay results demonstrated that these inhibitors partially attenuated the tumor suppressive effects of CaCO_3_@CM-OA in both HT29 and Patu-8988T cells, suggesting that activation of necroptosis, apoptosis, and autophagy pathways collectively contribute to its antitumor activity (Fig. S6E).

Moreover, the efficacy of diverse tumor immunotherapy strategies, particularly oncolytic virotherapy, depends on their ability to stimulate the host's intrinsic antitumor immune response. These therapeutic modalities promote immunogenic cell death, enhancing tumor-associated antigen presentation and stimulating the release of damage-associated molecular patterns (DAMPs), including ATP, HMGB1, and calreticulin (CRT), thereby facilitating immune cell recruitment and activation. As shown in Fig. S7A, Immunofluorescence analysis revealed that CaCO_3_@CM-OA treatment markedly promote calreticulin (CRT) exposure and HMGB1 translocation in HT29 cells, with comparable effects observed in Patu-8988T cells (Fig. S7B). Besides, treatment with OA, CM-OA, or CaCO_3_@CM-OA induced differential ATP release from both HT29 and Patu-8988T cells (Fig. S7C).

### 3.8 CaCO_3_@CM-OA exhibits potent therapeutic efficacy against colorectal cancer in vivo

**Fig. 5:**
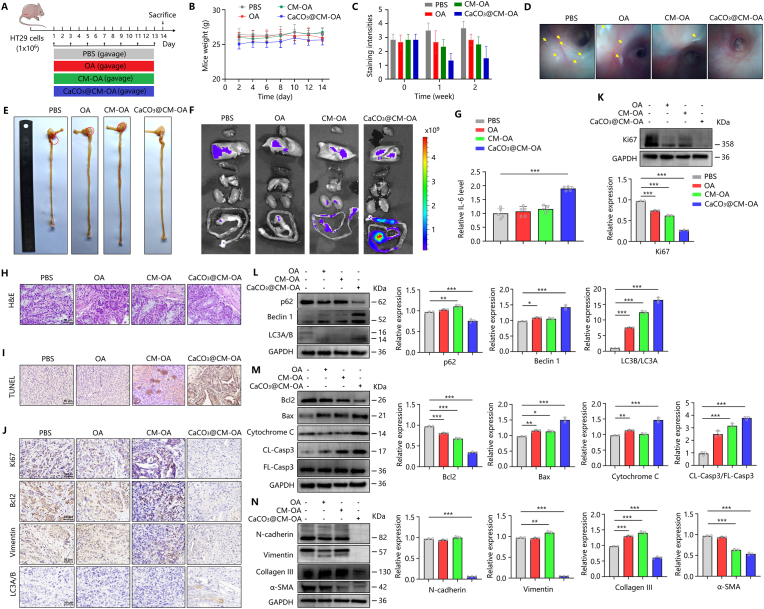
CaCO_3_@CM-OA suppresses CRC growth and epithelial–mesenchymal transition (EMT). (A) Mice bearing orthotopic colorectal tumors received oral administration every other day for 14 days. (B) Body weight monitoring. (C) Fecal occult blood test results. (D) Endoscopic images of the intestinal tract. (E) Tumor images from each treatment group. (F) Fluorescence imaging of major organs and colorectal tissue. (G) Serum IL-6 levels in each group. (H) H&E staining of tumors. (I) TUNEL assay on paraffin-embedded tumors. (J) IHC staining of Ki67, Bcl2, Vimentin, and LC3A/B. (K–N) Western blot analyses of Ki67, P62, Beclin1, LC3A/B, Bcl2, Bax, Cytochrome-c, N-cadherin, Vimentin, Collagen III, and α-SMA in tumor tissues. Data are presented as mean ± SD. ∗P < 0.05, ∗∗P < 0.01, ∗∗∗P < 0.001.

To investigate the anti-colorectal cancer effects of CaCO_3_@CM-OA in vivo, we further established an orthotopic model of colorectal cancer (Fig. 5A). Different treatments were administered via gavage for 14 days, and body weight was detected every other day (Fig. 5B). At the same time, we collected mouse feces every week to detect occult blood (Fig. 5C). Additionally, we observed intestinal lesions of mice by endoscopy (Fig. 5D). Compared to control groups such as OA, CaCO_3_@CM-OA treatment effectively reduced fecal occult blood positivity and intestinal lesions.

At the end treatment, the mice were sacrificed, and the tumors were collected (Fig. 5E). The distribution of oncolytic adenovirus in vivo was detected using a small animal imager (Fig. 5F). The results showed that OA, CM-OA, and CaCO_3_@CM-OA (all labeled with CY5-NHS) were enriched in the cecum and colorectal regions, with higher fluorescence intensity in the CaCO_3_@CM-OA treatment group. This suggests that CaCO_3_@CM-OA has good tumor targeting ability and resistance to digestive clearance. Additionally, CaCO_3_@CM-OA seems to be able to increase the level of IL-6 more significantly (Fig. 5G).

HE staining of tumor tissues revealed that the density and number of colorectal cancer cells were significantly inhibited by CaCO_3_@CM-OA (Fig. 5H). TUNEL staining also demonstrated the effect of CaCO_3_@CM-OA on promoting tumor apoptosis (Fig. 5I). Furthermore, we detected the expression of Ki67 by western blot. The results showed that CaCO_3_@CM-OA effectively inhibited tumor proliferation in vivo (Fig. 5K). Detection of protein expressions such as Bcl2 and Bax also indicated that CaCO3@CM-OA treatment significantly activated apoptosis signals in colorectal cancer cells (Fig. 5M). As Fig. 5L showed that CaCO_3_@CM-OA treatment increased the expression of Beclin 1 and LC3B/LC3A while decreasing p62 expression, suggesting that CaCO_3_@CM-OA activated autophagy pathway. Consistent with the results in vitro, the expression of N-cadherin and Vimentin were also efficiently suppressed by CaCO_3_@CM-OA in vivo (Fig. 5N). Immunohistochemical results for Ki67, Bcl2, Vimentin, and LC3A/B was performed, and showed the same trend (Fig. 5J). Besides, analysis of serum transaminase (Fig. S2A) and HE staining of organs such as the heart, liver, and spleen (Fig. S2B) demonstrated the CaCO_3_@CM-OA has good biosafety.

### 3.9 CaCO_3_@CM-OA exhibits potent therapeutic efficacy against pancreatic cancer in vivo

**Fig. 6:**
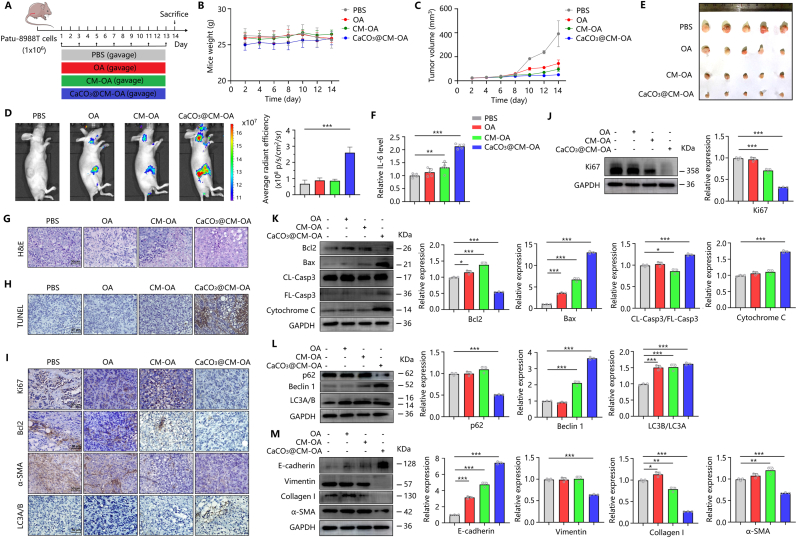
CaCO_3_@CM-OA inhibits pancreatic cancer (PCC) progression and EMT. (A) Mice bearing PCC xenografts were treated orally every other day for 14 days. (B) Body weight monitoring. (C) Tumor volume measurements. (D) Fluorescence imaging of tumors. (E) Tumor photographs. (F) Serum IL-6 levels. (G) H&E staining. (H) TUNEL assay. (I) IHC staining of Ki67, Bcl2, Vimentin, and LC3A/B. (J–M) Western blot results for Ki67, P62, Beclin1, LC3A/B, Bcl2, Bax, FL-Cas3, CL-Cas3, Cytochrome-c, E-cadherin, Vimentin, Collagen I, and α-SMA. Data are presented as mean ± SD. ∗P < 0.05, ∗∗P < 0.01, ∗∗∗P < 0.001.

To evaluate the anti-pancreatic cancer effect of CaCO_3_@CM-OA in vivo, we constructed a pancreatic cancer xenograft model. Tumor-bearing mice were treated with PBS, OA, CM-OA, and CaCO_3_@CM-OA (all labeled with CY5-NHS) for 14 days, as previously mentioned (Fig. 6A). The weight and tumor volume of the mice were regularly recorded (Fig. 6B and C). At the end of treatment, the distribution of oncolytic viruses in each group was visualized using a small animal imager. Compare with control group, the fluorescence of labeled virus was mainly enriched in tumor of the mice treated with CaCO_3_@CM-OA. This suggests that CaCO_3_@CM-OA exhibits good tumor targeting ability and resistance to immune clearance (Fig. 6D). The volume of tumors in the CaCO_3_@CM-OA treatment group were also significantly decreased (Fig. 6E). Detection of IL-6 levels in serum indicated that CaCO_3_@CM-OA treatment could significantly activate the inflammatory response in vivo, which aids in inhibiting tumor progression (Fig. 6F).

Hematoxylin and eosin (HE) staining of tumor revealed that the density and number of pancreatic cancer cells were significantly inhibited by CaCO_3_@CM-OA (Fig. 6G). TUNEL staining of tumor tissue also demonstrated that CaCO_3_@CM-OA significantly promoted tumor cell apoptosis (Fig. 6H). By Western blot, we detected the expression of Ki67 in tumor, showing that CaCO_3_@CM-OA effectively inhibited tumor cell proliferation in vivo (Fig. 6J). In addition, the expression of Bcl2 and Bax also indicated that CaCO_3_@CM-OA treatment significantly activated apoptosis signals in pancreatic cancer cells (Fig. 6K). As shown in Fig. 6L, CaCO_3_@CM-OA treatment also increased the expression of Beclin 1 and LC3B/LC3A, indicating the activation of autophagy signaling pathway Detection of N-cadherin and Vimentin showed that CaCO_3_@CM-OA also inhibited the epithelial-mesenchymal transition (EMT) process of pancreatic cancer in vivo (Fig. 6M). Immunohistochemical results of Ki67, Bcl2, Vimentin, and LC3A/B exhibit the same trend, demonstrating that CaCO_3_@CM-OA also exerted a good anti-pancreatic cancer effect in vivo (Fig. 6I).

### 3.10 CaCO_3_@CM-OA exerts antitumor effects potentially via targeting CDC27, ZMYND10, ST7, RHBDD2, and SNX11

**Fig. 7:**
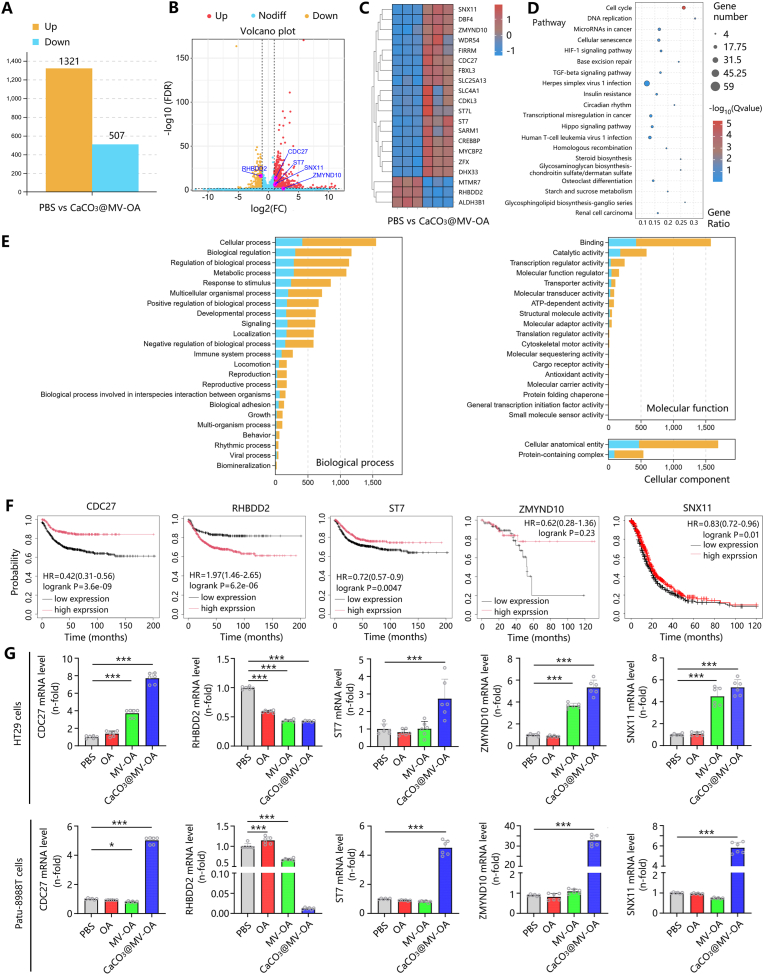
Potential molecular targets associated with the anti-tumor effects of CaCO_3_@CM-OA. (A) CaCO_3_@CM-OA induces differential gene expression in tumor cells. (B) Volcano plot. (C) Heatmap of the top 20 differentially expressed genes. (D, E) KEGG and GO enrichment analyses. (F) Survival analysis of CDC27, RHBDD2, ST7, ZMYND10, and SNX11. (G) mRNA expression of these genes across treatment groups. Data are presented as mean ± SD. ∗P < 0.05, ∗∗P < 0.01, ∗∗∗P < 0.001.

Transcriptome sequencing analysis revealed that the CaCO_3_@CM-OA induced significant differential expression of multiple genes in tumor cells (Fig. 7A). Among these, the most prominently altered genes included CDC27, ZMYND10, ST7, RHBDD2, and SNX11 (Fig. 7B and C). KEGG enrichment analysis demonstrated that CaCO_3_@CM-OA modulated tumor cell pathways associated with cell cycle, DNA replication, and cellular senescence (Fig. 7D). GO enrichment results demonstrated that CaCO_3_@CM-OA induced alterations in immune system processes, biological adhesion, and viral processes within tumor cells (Fig. 7E). Subsequently, survival analysis was performed on these genes (Fig. 7F), and their expression changes in HT29 and Patu-8988T cells were validated via qRT-PCR (Fig. 7G). The results indicated that the differential expression of these genes significantly influenced overall patient survival rates.

## 4 Discussion

Colorectal cancer (CRC) and pancreatic cancer (PCC) are aggressive gastrointestinal malignancies characterized by high rates of metastasis and poor prognosis. Traditional treatments such as surgery, chemotherapy, and radiotherapy remain the clinical mainstay [27,28]. However, their efficacy is often limited due to late-stage diagnosis, drug resistance, and tumor heterogeneity [29,30]. Oncolytic virotherapy, particularly oncolytic adenoviruses, has emerged as a promising therapeutic intervention, and has been initially applied in the clinical treatment of liver cancer and melanoma [[31], [32], [33]]. Despite their potential, Oncolytic virotherapy face challenges including immune clearance, poor infectivity in tumors with low specific receptors expression, and administration limitations [[34], [35], [36], [37], [38]]. In this study, we developed a novel biomineralized oncolytic adenovirus (CaCO_3_@CM-OA) to enhance oncolytic adenovirus stability, immune evasion, and oral bioavailability.

Uncontrolled proliferation is a hallmark of cancer, often driven by dysregulated cell cycle machinery [39]. The abnormal cell cycle regulation in tumor cells manifests primarily through three mechanisms: ① Overactivation of positive regulatory factors, such as overexpression of Cyclins and Cyclin-dependent kinases (CDKs); ② Dysfunction of negative regulatory factors, including suppressed expression of Cyclin-dependent kinase inhibitors (CKIs); ③ Failure of the quality control system, specifically, abnormalities in cell cycle checkpoint functions. TP53, a crucial tumor suppressor gene, often exhibits abnormal mutations and loss of function in most tumors [40]. The main function of p53, the expression product of TP53, is to induce the expression of its downstream target genes through a series of phosphorylation events. This process participates in DNA repair and cell cycle arrest, maintaining genomic stability and suppressing tumorigenesis. CDKN1A is one of the important downstream target genes of p53. Activated p53 binds to elements in the CDKN1A promoter and activates its transcription. The expression product of CDKN1A, p21, can inhibit the Cyclin-CDK pairs involved in the cell cycle process, thereby inducing cell cycle arrest. Simultaneously, activated p21 can downregulate the transcription of cell cycle genes by promoting the formation of a complex between Rb (Retinoblastoma protein) and the E2F transcription factor family. Our results showed that CaCO_3_@CM-OA significantly inhibited proliferation in CRC and PCC cells, likely via induction of G2/M phase arrest. This was evidenced by elevated phosphorylation of p53 and upregulation of p21, key regulators of cell cycle checkpoints. Concurrently, accumulation of Cyclin B1 and suppression of proliferation marker Ki67 support this arrest. These findings suggest that CaCO_3_@CM-OA disrupts cell cycle progression through p53/p21-mediated mechanisms, thereby inhibiting tumor growth.

Apoptosis resistance contributes to tumor survival and therapy failure. The anti-apoptotic protein Bcl2 primarily functions as an integral membrane protein in organelles such as mitochondria, interacting with other Bcl2 family members to maintain the stability of the mitochondrial membrane [41,42]. Bax, also a member of the Bcl2 family, induces the release of apoptotic factors such as cytochrome-c and initiates a cascade of caspase activation to exert a pro-apoptotic effect [43,44].

In our study, CaCO_3_@CM-OA treatment significantly increased apoptosis, as indicated by flow cytometry and enhanced expression of pro-apoptotic markers Bax, Cytochrome *c*, and cleaved-Caspase 3, alongside suppression of anti-apoptotic Bcl-2. Notably, calcium carbonate, a component of the delivery system, may enhance apoptosis by increasing extracellular calcium ion levels and activating calcium-sensing pathways. According to our results, apoptosis rate in the CaCO_3_@CM-OA group is significantly higher than control group, we hypothesize that the more significant apoptotic signal not only related to CaCO_3_@CM-OA enhances OA's infectivity towards tumor cells. A clinical trial has demonstrated that calcium supplementation can reduce colorectal cancer markers and benefit patients with colorectal adenocarcinoma [45,46]. These findings highlight the synergistic role of CaCO_3_@CM-OA in promoting mitochondrial-mediated apoptosis.

Autophagy, a lysosomal degradation pathway, plays dual roles in cancer. Experimental results have demonstrated that certain viruses can induce autophagy after infecting tumor cells, thereby inhibiting tumor growth [44]. Current research suggests that the mechanisms by which oncolytic viruses induce autophagy and enhance antitumor effects may be multifaceted. Firstly, oncolytic viruses can impede autophagy-inhibiting signals such as AKT/mTOR and Bcl2 [47,48], increasing the formation of autophagosomes. Additionally, studies have confirmed that oncolytic viruses can utilize autophagosomes as replication sites, facilitating their own replication [49]. Lastly, autophagy can also enhance the presentation of tumor cell-associated antigens regulating specific immune responses [50]. In our study, CaCO_3_@CM-OA induced autophagy as evidenced by increased expression of Beclin 1 and LC3B/LC3A, and reduced levels of autophagic flux marker p62. This suggests that autophagy contributes to the enhanced tumoricidal effect of CaCO_3_@CM-OA.

Necroptosis, another form of regulated cell death, was also activated following CaCO_3_@CM-OA treatment, as indicated by elevated phosphorylation of MLKL. Necroptosis promotes immunogenic tumor cell death, enhancing immune recognition via damage-associated molecular patterns (DAMPs) [51,52]. Moreover, our study demonstrated upregulation of ATP release, CRT exposure, and HMGB1 translocation [53,54], confirming that CaCO_3_@CM-OA activates immunogenic cell death pathways, thereby potentially boosting antitumor immunity.

Given that both of colorectal cancer and pancreatic cancer are tumor types prone to distant metastasis, with epithelial-mesenchymal transition (EMT) being a pivotal factor in this process [55,56], we examined its modulation by CaCO_3_@CM-OA. Through in *vitro* and in *vivo* experiments, our results revealed that CaCO_3_@CM-OA significantly reduced migration and invasion of CRC and PCC cells and suppressed EMT marker expression, including N-cadherin, Vimentin, and α-SMA. This inhibition of EMT may further contribute to the observed antimetastatic effects.

To explore potential molecular targets, we performed transcriptomic profiling post-treatment. Notably, RHBDD2, a gene linked to anti-apoptotic signaling, was significantly downregulated, suggesting a novel mechanism underlying CaCO_3_@CM-OA-induced apoptosis. Changes in ZMYND10, CDC27, and ST7 expression further implicate this virus in cell cycle and proliferation regulation. Interestingly, upregulation of SNX11—a gene associated with endocytosis—may enhance viral entry and efficacy.

Recent studies have demonstrated the therapeutic benefits of biomineralized oncolytic adenovirus in various cancers through intratumoral or intravenous routes [57,58]. However, our work is distinct in its design of an orally administrable CaCO_3_@CM-OA capable of resisting digestive degradation and immune clearance. In addition, the lysosomal escape capability of CaCO_3_@CM-OA may also contribute to its enhanced intracellular delivery efficiency. Previous studies have demonstrated that CaCO_3_ nanoparticles can facilitate endosomal and lysosomal escape through a pH-triggered decomposition process, generating CO_2_ and Ca^2+^ that increase osmotic pressure and disrupt the endo/lysosomal membrane [59]. This innovation expands the application scope of oncolytic adenovirus, particularly for deep-seated tumors such as CRC and PCC, which are less accessible via traditional injection-based methods. Furthermore, we systematically investigated the impact of oncolytic adenovirus entry into tumor cells on cell proliferation, metastasis, and the activation of cell death pathways such as apoptosis, autophagy, and necrosis.

In conclusion, CaCO_3_@CM-OA offers a multifunctional approach to cancer therapy by combining improved delivery with potent antitumor mechanisms. It promotes tumor cell death through apoptosis, autophagy, and necroptosis, suppresses proliferation and EMT, and enhances immunogenicity. Although our findings are promising, further investigation is warranted to validate its safety and therapeutic potential in clinical settings. This study provides a data reference for future development of biomineralized oncolytic virotherapies.

## 5 Conclusion

In this study, we developed a novel biomineralized oncolytic adenovirus with enhanced resistance to digestive enzymes and neutralizing antibodies, significantly improving tumor targeting and infection efficiency. In colorectal and pancreatic cancer cells, the virus inhibited cell proliferation by inducing G2/M phase arrest, potentially mediated by increased p53 phosphorylation and p21 upregulation. It also suppressed epithelial-mesenchymal transition (EMT), as evidenced by the downregulation of mesenchymal markers such as N-cadherin, Vimentin, and α-SMA.

Moreover, the virus exerted potent cytotoxicity through the activation of multiple cell death pathways, including mitochondrial-mediated apoptosis, autophagy, and necroptosis. These effects were accompanied by classical hallmarks of immunogenic cell death, such as ATP release, calreticulin exposure, and HMGB1 translocation, indicating robust activation of antitumor immunity. Furthermore, transcriptomic profiling further revealed that the virus downregulated key pro-survival genes like RHBDD2, while modulating genes related to cell proliferation (e.g., ZMYND10, CDC27, ST7) and endocytosis (SNX11), offering insights into its multifaceted mechanisms of action.

In conclusion, this biomineralized oncolytic adenovirus demonstrates a multi-pronged antitumor effect by inducing diverse forms of regulated cell death and enhancing immune responses. The identification of potential gene targets provides new directions for future mechanistic studies and lays the groundwork for clinical translation of this therapeutic strategy.

## CRediT authorship contribution statement

**Zujian Hu:** Writing – review & editing, Writing – original draft, Software, Methodology, Formal analysis, Data curation. **Yining Sun:** Methodology, Formal analysis. **Shenlei Yu:** Software, Methodology, Formal analysis. **Fan Zheng:** Resources, Methodology, Formal analysis. **Zhuo Yan:** Software, Investigation, Formal analysis. **Ning Lu:** Software, Resources, Investigation. **Luyi Ye:** Validation, Methodology. **Shanshan Yuan:** Software, Methodology, Investigation. **Yuting Zhu:** Software, Methodology, Formal analysis. **Junjie Deng:** Writing – review & editing, Writing – original draft, Supervision. **Jilong Wang:** Supervision, Funding acquisition. **Yongheng Bai:** Writing – review & editing, Writing – original draft, Funding acquisition.

## Declaration of competing interest

The authors declare that they have no known competing financial interests or personal relationships that could have appeared to influence the work reported in this paper.

## Data Availability

Data will be made available on request.
