# Supplementary material for: Enhanced oral nanomedicine utilizing biomineralized oncolytic virus for synergistic gastrointestinal cancer therapy
Source: Mater Today Bio. 2025 Nov 21;35:102583. doi: 10.1016/j.mtbio.2025.102583 (PMC12689225; doi:10.1016/j.mtbio.2025.102583)
Supplement: Multimedia component 1 [file mmc1.docx]

Enhanced oral nanomedicine utilizing biomineralized oncolytic virus for synergistic gastrointestinal cancer therapy

Zujian Hu^a,b,c^, Yining Sun^a^, Shenlei Yu^a^, Fan Zheng^a^, Zhuo Yan^c,d^, Ning Lu^c,d^, Luyi Ye^c,d^, Shanshan Yuan^c,d^, Yuting Zhu^c,d^, Junjie Deng^c,d^*, Jilong Wang^c,d,^*, Yongheng Bai^a,c,^*

^a^ Zhejiang Key Laboratory of Intelligent Cancer Biomarker Discovery and Translation, The First Affiliated Hospital, Wenzhou Medical University, Wenzhou 325035, China

^b^ National Clinical Research Center for Ocular Diseases, Eye Hospital, Wenzhou Medical University, Wenzhou, 325027, China

^c^ Joint Centre of Translational Medicine, The First Affiliated Hospital of Wenzhou Medical University, Wenzhou Medical University, Wenzhou, Zhejiang 325000, China

^d^ Joint Centre of Translational Medicine, Wenzhou Institute, University of Chinese Academy of Sciences, Wenzhou, Zhejiang 325000, China

* Corresponding authors: wzbyh@wmu.edu.cn (Y. Bai), wangjilong@ucas.ac.cn (J. Wang), j.deng@ucas.ac.cn (J. Deng)

**Supplementary Text**

*Immunofluorescence Staining*

HT29 and Patu-8988T cells were seeded in 24-well plates at a density of 5×10⁴ cells/well and treated with PBS, OA, CM-OA, or CaCO₃@CM-OA for 48 h. Cells were then fixed with 4% paraformaldehyde (Solarbio, China) for 30 min and permeabilized with 0.1% Triton X-100 (Solarbio, China) for 10 min. After blocking with normal goat serum, cells were incubated with the appropriate primary antibody overnight at 4 °C. The next day, the slides were incubated with a fluorescent secondary antibody for 1 h at 37°C (in the dark), and the nuclei were stained with DAPI.

*Histological Examination*

In order to evaluate the potential toxicity of CaCO₃@CM-OA to the organ., tumor tissues were fixed in 4% paraformaldehyde, embedded in paraffin, and sectioned at 4 μm thickness. Sections were deparaffinized, rehydrated, and stained with Hematoxylin-Eosin (H&E) using a commercial kit (Solarbio, G1121) according to the manufacturer's instructions.

*Quantitative Real-Time PCR (qRT-PCR)*

Total RNA was extracted from cells or tissues using TRIzol reagent (GLPBIO, Shanghai, China) following the manufacturer's protocol. One microgram of RNA was reverse transcribed into cDNA using the HiScript III RT SuperMix for qPCR kit (Vazyme, Nanjing, China). qRT-PCR was performed using SYBR Green Mix (Vazyme) on a QuantStudio 5 Real-Time PCR System (Thermo Fisher Scientific, USA). Relative mRNA expression levels were calculated using the 2^−ΔΔCt method.

*Western Blot Analysis*

Total protein was extracted using RIPA lysis buffer (Beyotime, Shanghai, China), separated by 12% SDS-PAGE, and transferred onto PVDF membranes. Membranes were blocked with 5% skim milk and incubated overnight at 4 °C with specific primary antibodies, followed by incubation with HRP-conjugated secondary antibodies for 1 h at room temperature. Protein bands were visualized using enhanced chemiluminescence reagents (ECL, A38554, Thermo Fisher Scientific, USA).

*ATP Assay*

ATP levels were quantified using an ATP Assay Kit (Beyotime, S0026) according to the manufacturer's instructions. After preparation of the working solution, samples and standards were added to a 96-well plate, and relative light units (RLU) were measured using a Varioskan LUX multimode plate reader. ATP concentrations were calculated from a standard curve.

*Lactate Dehydrogenase (LDH) Assay*

LDH release was assessed using an LDH Cytotoxicity Assay Kit (Beyotime, C0017) following the manufacturer's protocol. Briefly, 120 μL of cell culture supernatant was added to a 96-well plate, followed by 60 μL of LDH detection reagent. The mixture was incubated in the dark at room temperature for 30 min with gentle shaking. Absorbance was measured at 490 nm using a microplate reader.

**
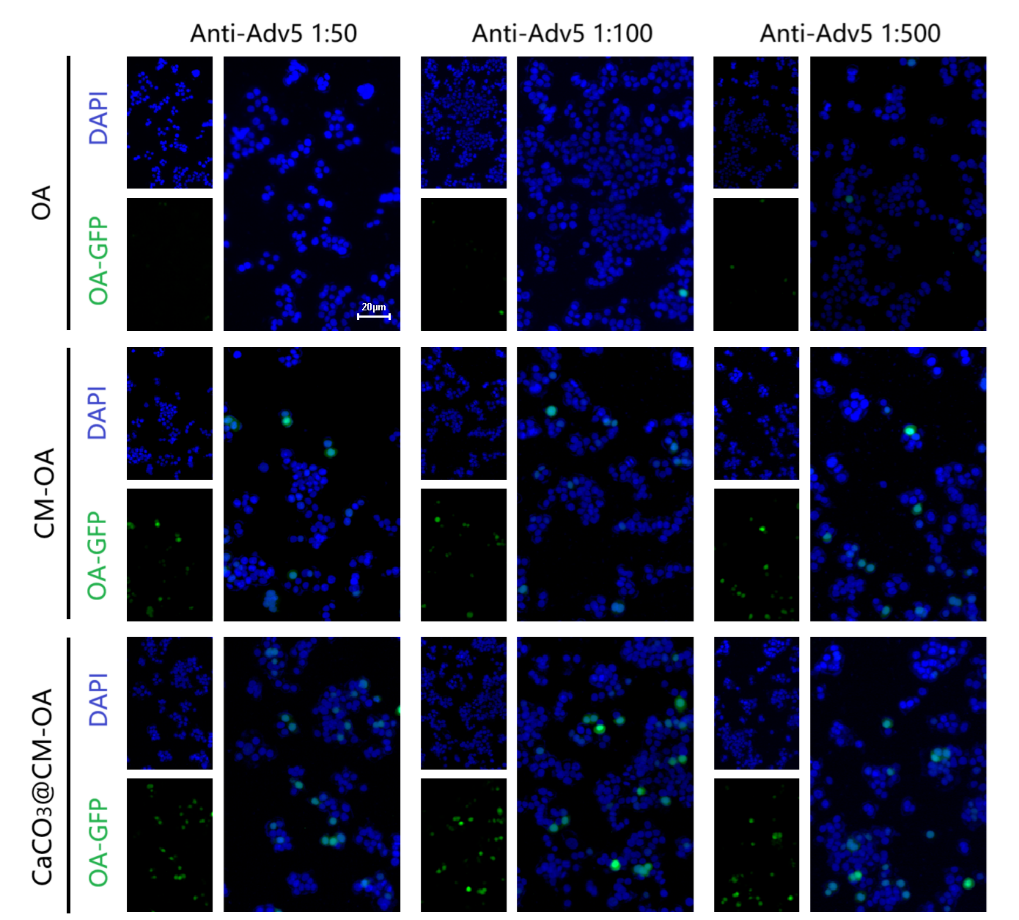
**

**Fig. S1.** Fluorescence images of tumor cells infected by OA, CM-OA, and CaCO_3_@CM-OA after treatment with various titers of adenovirus type 5 hexon antibody.

**
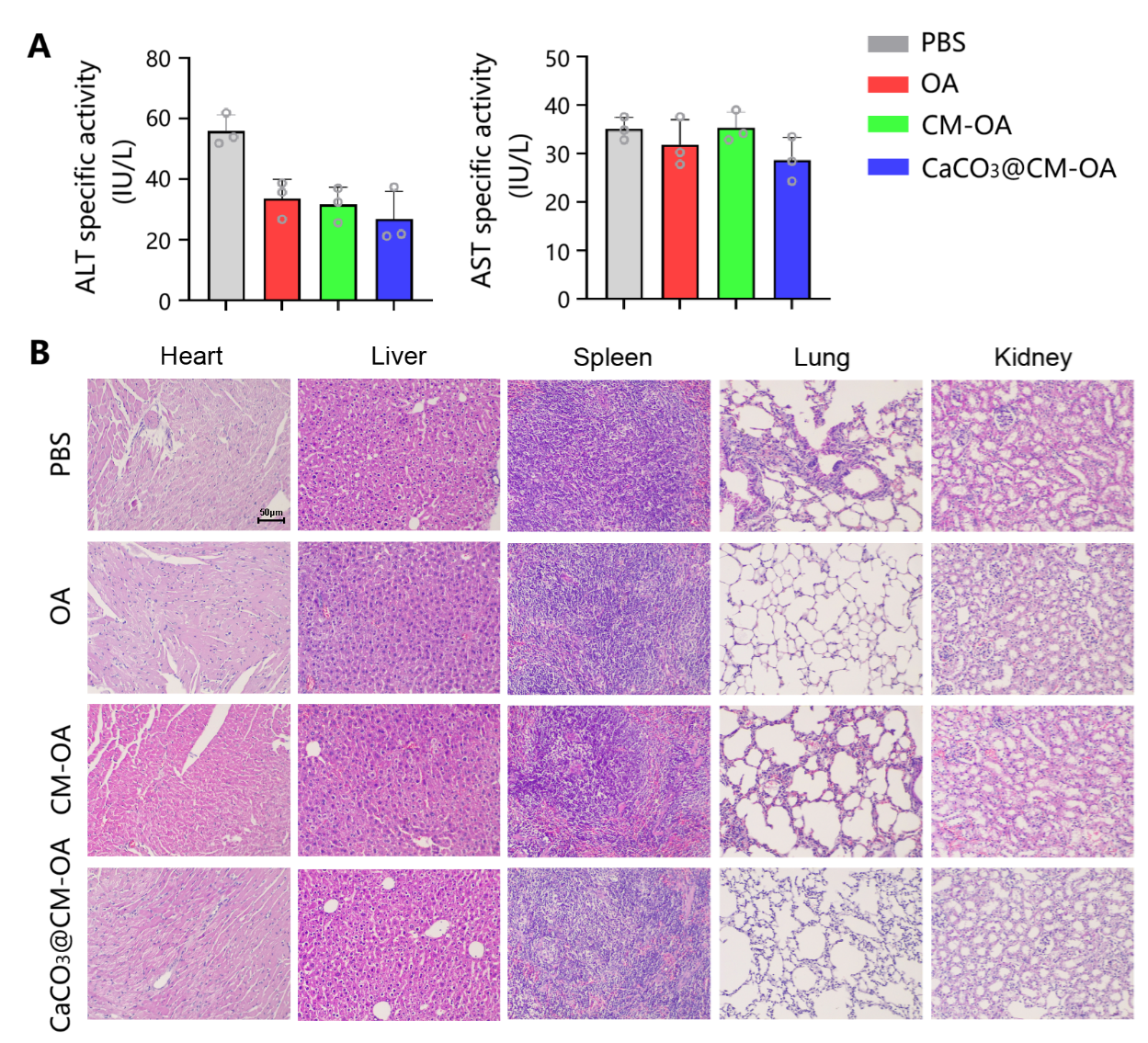
**

**Fig. S2.** (A) Serum ALT and AST levels in mice from each group. (B) H&E staining of major organs.

**
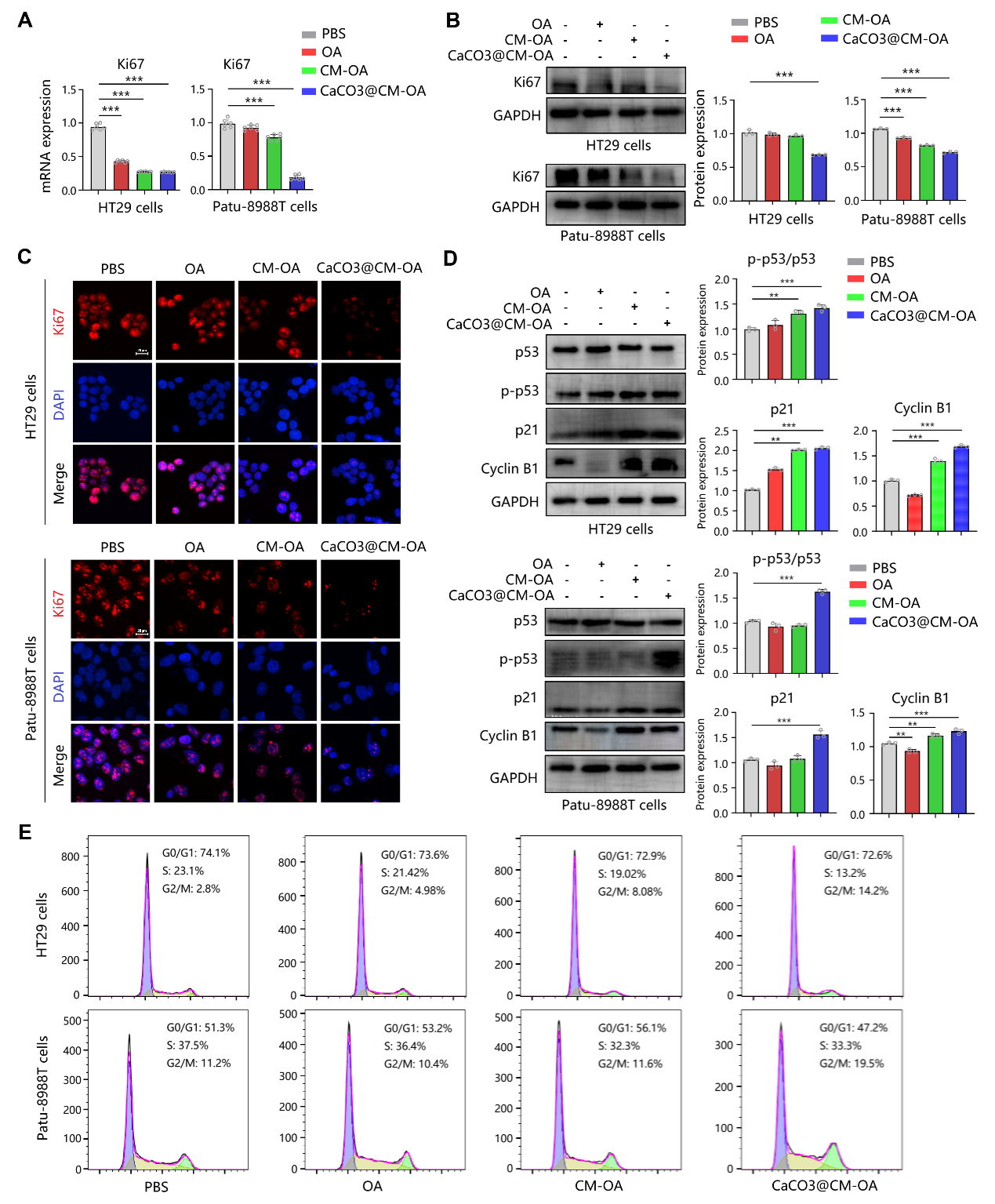
**

**Fig. S3.** (A) mRNA expression of Ki67 in HT29 and Patu-8988T cells. (B) Western blot and quantification of Ki67. (C) Immunofluorescent staining of Ki67. (D) Western blot of p53, p-p53, p21, and cyclinB1. (E) Cell cycle distribution via flow cytometry. Data are presented as mean ± SD. *P<0.05, **P<0.01, ***P <0.001.


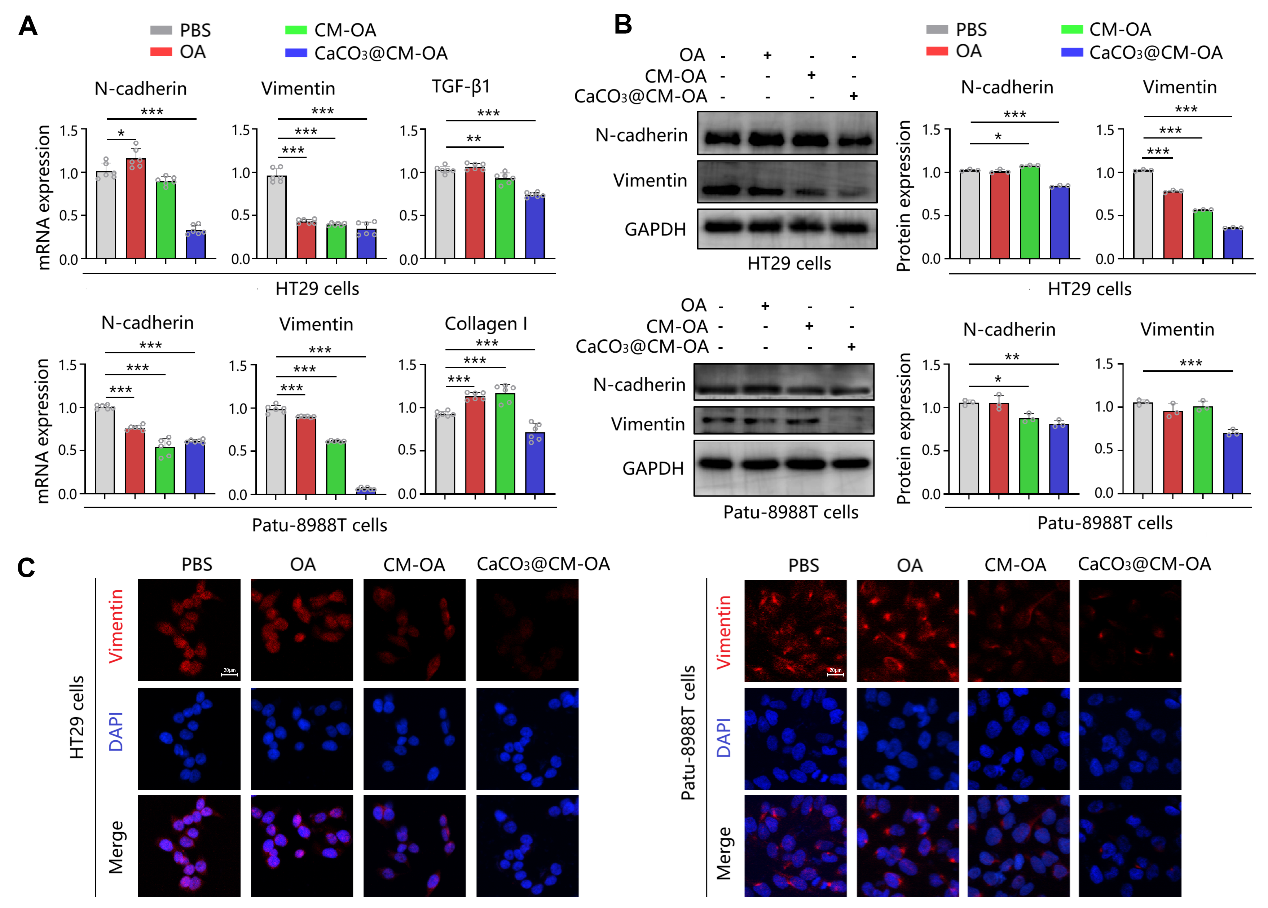


**Fig. S4.** (A) mRNA expression of N-cadherin, Vimentin, TGF-β1, and Collagen I. (B) Western blot and quantification of N-cadherin and Vimentin. (C) Immunofluorescent staining of Vimentin. Data are presented as mean ± SD. *P<0.05, **P<0.01, ***P <0.001.


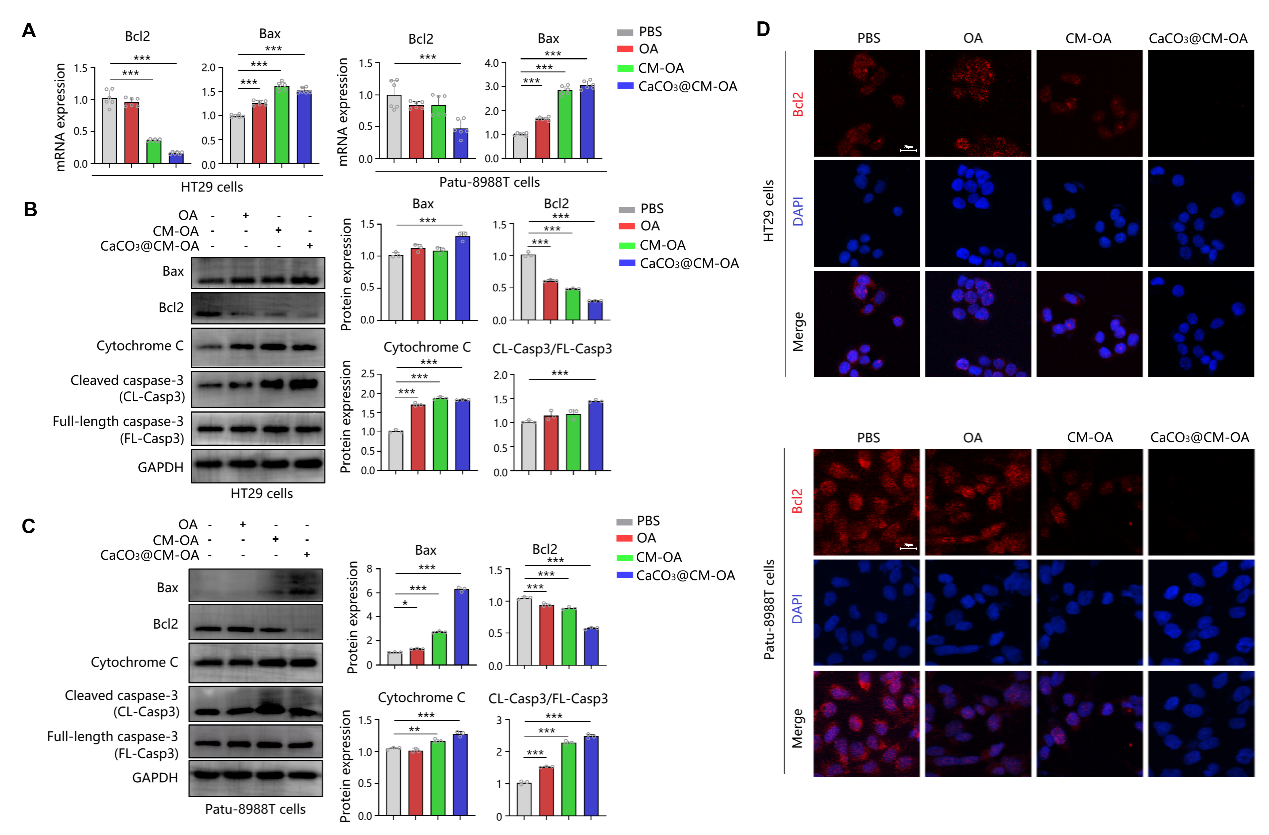


**Fig. S5.** (A) mRNA levels of Bcl2 and Bax. (B, C) Western blot and analysis of Bcl2, Bax, cytochrome-c, FL-Cas3, and CL-Cas3. (D) Immunofluorescent staining of Bcl2. Data are presented as mean ± SD. *P<0.05, **P<0.01, ***P <0.001.

**
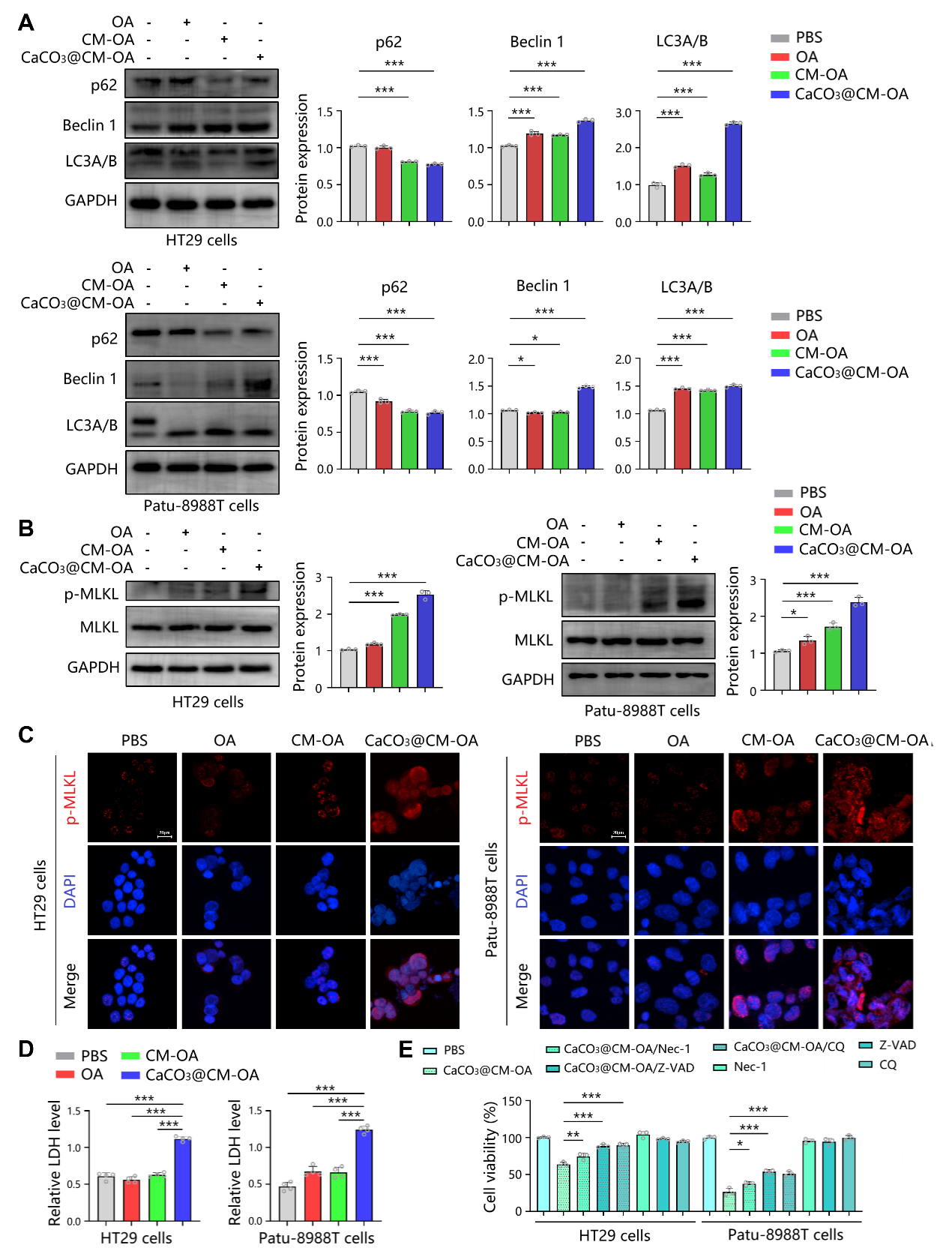
**

**Fig. S6.** (A) Western blot and analysis of p62, Beclin1, and LC3A/B. (B) Western blot of MLKL and p-MLKL. (C) Immunofluorescent staining of p-MLKL. (D) Relative LDH levels in culture supernatant. (E) CCK-8 assay for viability with or without CaCO3@CM-OA and inhibitors (NEC-1, Z-VAD, CQ). Data are presented as mean ± SD. *P<0.05, **P<0.01, ***P <0.001.

**
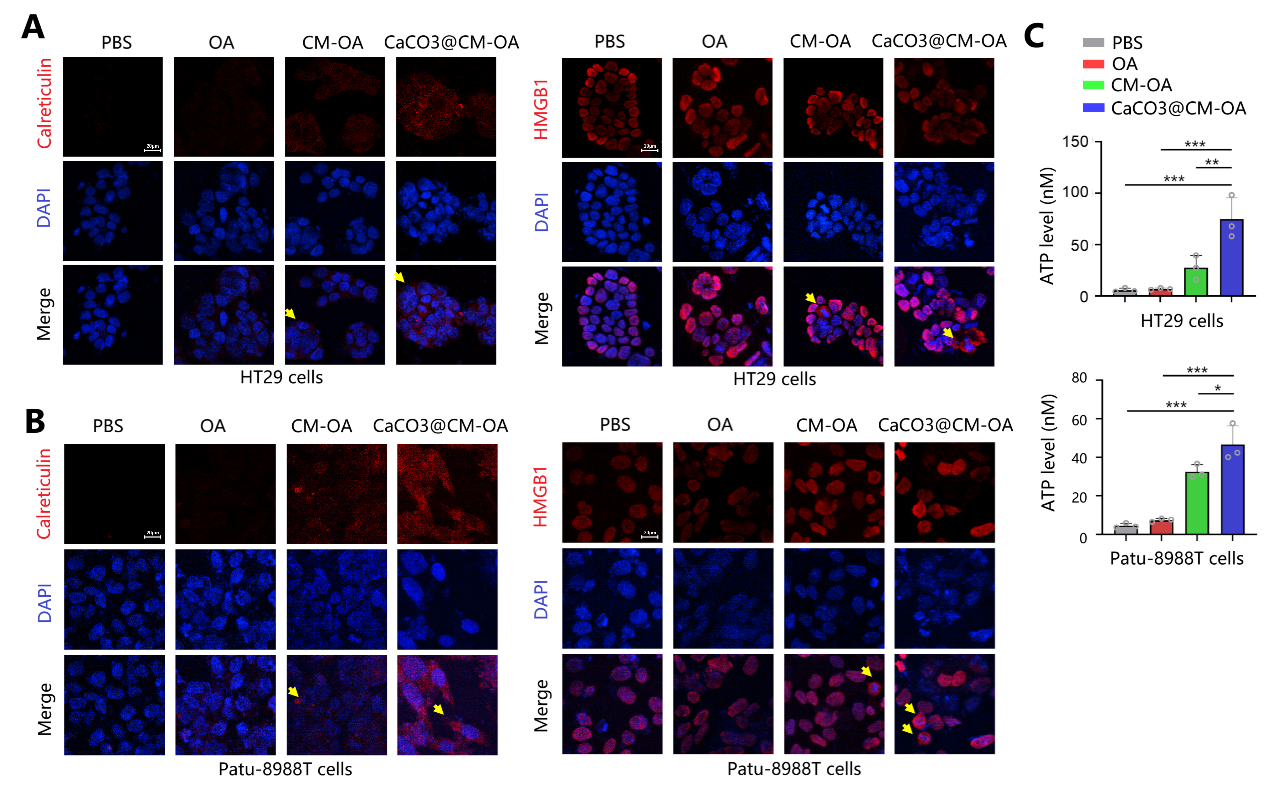
**

**Fig. S7.** (A) Immunofluorescent staining of Calreticulin. (B) HMGB1 staining. (C) Relative ATP levels in culture supernatant. Data are presented as mean ± SD. *P<0.05, **P<0.01, ***P <0.001.
